# Biomolecules Interacting with Long Noncoding RNAs

**DOI:** 10.3390/biology14040442

**Published:** 2025-04-19

**Authors:** Hidenori Tani

**Affiliations:** Department of Health Pharmacy, Yokohama University of Pharmacy, 601 Matano, Totsuka, Yokohama 245-0066, Japan; hidenori.tani@yok.hamayaku.ac.jp

**Keywords:** long noncoding RNA, gene regulation, cellular function, RNA–DNA triplexes, miRNA sponge

## Abstract

This review explores the complex interactions between long noncoding RNAs (lncRNAs) and other biomolecules, highlighting their pivotal roles in gene regulation and cellular function. LncRNAs, defined as RNA transcripts exceeding 200 nucleotides without encoding proteins, are involved in diverse biological processes, from embryogenesis to pathogenesis. They interact with DNA through mechanisms like triplex structure formation, influencing chromatin organization and gene expression. LncRNAs also modulate RNA-mediated processes, including mRNA stability, translational control, and splicing regulation. Their versatility stems from their forming of complex structures that enable interactions with various biomolecules. This review synthesizes current knowledge on lncRNA functions, discusses emerging roles in development and disease, and evaluates potential applications in diagnostics and therapeutics. By examining lncRNA interactions, it provides insights into the intricate regulatory networks governing cellular processes, underscoring the importance of lncRNAs in molecular biology. Unlike the majority of previous reviews that primarily focused on individual aspects of lncRNA biology, this comprehensive review uniquely integrates structural, functional, and mechanistic perspectives on lncRNA interactions across diverse biomolecules. Additionally, this review critically evaluates cutting-edge methodologies for studying lncRNA interactions, bridges fundamental molecular mechanisms with potential clinical applications, and highlights their potential.

## Simple Summary

Long noncoding RNAs are a type of genetic material that do not produce proteins but play important roles in controlling how genes work inside our cells. This review explains how these molecules interact with DNA, other RNAs, and proteins to influence many biological processes, such as development, cell identity, and disease. For example, long noncoding RNAs can help organize the structure of our genetic material, regulate which genes are turned on or off, and affect how other RNAs are processed or broken down. They also act as scaffolds, bringing together different molecules to form specialized structures inside cells. Recent research has shown that these interactions are crucial for normal development and are involved in diseases like cancer and neurodegenerative disorders. Understanding how long noncoding RNAs function could lead to new ways to diagnose diseases and develop targeted treatments. This review brings together the latest findings and highlights new technologies that are helping scientists study these complex molecules, offering insights that may benefit medicine and society in the future.

## 1. Introduction

The field of molecular biology has undergone a paradigm shift with the discovery of long noncoding RNAs (lncRNAs) [1]. These enigmatic molecules, defined as RNA transcripts exceeding 200 nucleotides in length that do not encode proteins, have emerged as pivotal regulators in the intricate landscape of gene regulation and cellular function [2,3]. The significance of lncRNAs is profound, as they have been implicated in a diverse array of biological processes, from embryogenesis to pathogenesis, the flow of genetic information from DNA through RNA to protein. LncRNAs constitute a vast and heterogeneous class of transcripts, potentially outnumbering protein-coding genes in the human genome. In contrast to messenger RNAs (mRNAs), which serve as templates for protein synthesis, lncRNAs exert their functions through a multitude of mechanisms different from translation. This functional versatility has captivated the scientific community and opened novel avenues for elucidating the intricacies of gene regulation and cellular homeostasis. The definition of lncRNAs, while ostensibly straightforward, belies the complexity and heterogeneity of this RNA class. The 200-nucleotide threshold, although somewhat arbitrary, serves to distinguish lncRNAs from well-characterized short noncoding RNAs, such as microRNAs and small interfering RNAs. However, it is crucial to note that length alone does not determine functionality, and some lncRNAs may indeed encode small peptides, blurring the distinction between coding and non-coding transcripts [4,5]. The importance of lncRNAs is underscored by their evolutionary conservation and tissue-specific expression patterns. While the primary sequence of many lncRNAs may not be highly conserved across species, their genomic loci, secondary structures, and expression profiles often exhibit significant conservation, suggesting functional relevance [6,7]. Moreover, the exquisite regulation of lncRNA expression in distinct cell types and developmental stages indicates their critical roles in maintaining cellular identity and orchestrating complex biological processes [6].

One of the most intriguing aspects of lncRNA biology is the diverse array of functions these molecules can perform. Unlike proteins, which often possess well-defined structural domains that dictate their functions, lncRNAs exhibit remarkable functional plasticity [8]. This versatility stems from their ability to form complex secondary and tertiary structures, enabling them to interact with a wide range of biomolecules, including DNA, RNA, and proteins. Despite significant progress in lncRNA research, numerous challenges and unanswered questions persist. The mechanisms by which lncRNAs achieve specificity in their interactions with other biomolecules remain incompletely elucidated. The evolutionary significance of lncRNAs and their contribution to species-specific traits are active areas of investigation [6]. Furthermore, the potential redundancy and compensatory mechanisms among lncRNAs complicate functional studies and therapeutic interventions [9].

This comprehensive review aims to synthesize the current state of knowledge regarding lncRNAs and their interactions with other biomolecules. I will explore the diverse mechanisms through which lncRNAs exert their biological functions, from transcriptional regulation [10] to post-transcriptional control of gene expression [11] and cellular organization [12]. By examining the interplay between lncRNAs and various cellular components, I seek to provide insights into the complex regulatory networks that govern cellular processes. This review will encompass the latest advancements in lncRNA research, including novel techniques for studying lncRNA structure and function [13], emerging roles in development and disease [14], and potential applications in diagnostics and therapeutics [15]. I will critically evaluate the evidence supporting various lncRNA functions and discuss the challenges and controversies in the field. Additionally, I will highlight important areas for future research and the potential impact of lncRNA studies on our understanding of genome organization and gene regulation [16]. By offering a comprehensive overview of lncRNA biology, this review aims to support researchers, clinicians, and students in this rapidly evolving field. This article aims to inspire new research directions and foster interdisciplinary collaborations that will further elucidate the mysteries of these fascinating RNA molecules and their roles in health and disease. Moreover, this review focuses on the interactions between lncRNAs and biomolecules in mammalian systems, with a particular emphasis on their roles in gene regulation and cellular function. While lncRNAs in plants represent an intriguing area of research, they are beyond the scope of this article.

Despite significant advances in lncRNA research over the past decade, most existing reviews have focused on specific aspects of lncRNA biology, such as their roles in particular diseases, their involvement in specific cellular processes, or their potential as biomarkers. For instance, recent reviews have addressed lncRNAs in cancer progression, their functions in immune regulation, or their utility as diagnostic tools [1,2,3]. However, there remains a critical need for an integrated analysis that comprehensively examines the diverse interactions of lncRNAs with various biomolecules and connects these interactions to their biological functions and potential clinical applications.

While recent comprehensive reviews, such as the Consensus Statement by Mattick et al. (2023) [1], have provided an outstanding and up-to-date overview of lncRNA biology—including definitions, functions, molecular mechanisms, and current challenges—this review aims to complement and extend these works in several specific ways. First, I place particular emphasis on a detailed and critical analysis of the structural basis of lncRNA interactions, which, although discussed in previous reviews, remains relatively underrepresented. Second, I uniquely integrate emerging concepts, such as phase separation and biomolecular condensate formation, with traditional views of lncRNA function, providing a nuanced perspective on how lncRNAs organize cellular processes. Third, I offer a practical evaluation of the latest methodologies for studying lncRNA interactions, with actionable insights for experimental researchers. Finally, this review bridges fundamental molecular mechanisms with translational and clinical applications, highlighting the path from basic lncRNA research to therapeutic and diagnostic strategies. Through these focal points, my review seeks to provide added value and new perspectives to the existing literature.

## 2. Interactions with LncRNAs

### 2.1. LncRNA–DNA Interactions

Interactions between lncRNAs and DNA are central to gene regulation, chromatin organization [16], and genome– stability [17]. This section focuses on three key aspects: triplex structure formation, enhancer-associated lncRNAs, and chromatin structure regulation. Triplex structure formation is a remarkable phenomenon that occurs when lncRNAs interact with double-stranded DNA to form a three-stranded nucleic acid structure. This unique configuration, also known as an RNA–DNA triplex or RNA:DNA hybrid loop (R-loop), is characterized by the formation of Hoogsteen or reverse Hoogsteen base pairs between the RNA strand and the purine-rich strand of the DNA duplex [18]. The resulting structure can have profound effects on DNA topology, accessibility, and function [17].

**Figure 1:**
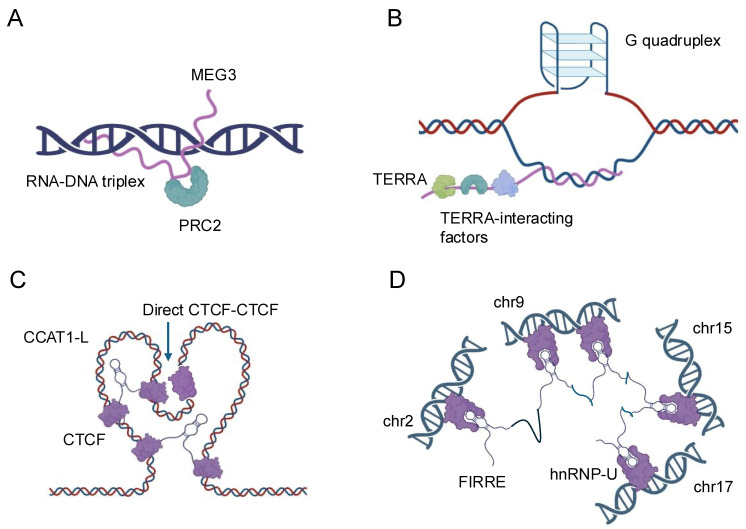
Schematic representation of the nuclear localization and functions of four representative lncRNAs. (**A**) MEG3: MEG3 is a nuclear-retained lncRNA that interacts with the Polycomb Repressive Complex 2 (PRC2) to regulate gene expression through chromatin modification. (**B**) TERRA: TERRA is a telomeric repeat-containing RNA that localizes to telomeres and interacts with various telomere-associated proteins, playing a crucial role in telomere maintenance and genome stability. (**C**) CCAT1-L: CCAT1-L is a nuclear lncRNA involved in the regulation of chromatin architecture and gene expression, particularly at the MYC locus, by mediating long-range chromatin interactions. (**D**) FIRRE: FIRRE is a lncRNA that localizes to the nuclear matrix and is implicated in the organization of higher-order chromatin structure by interacting with multiple genomic loci across different chromosomes.

The formation of RNA–DNA triplexes is sequence-specific and depends on the presence of poly-purine tracts, typically consisting of at least 10 consecutive purine bases, in the target DNA. These structures are typically formed by lncRNAs that contain complementary sequences to the target DNA region. Thus, lncRNAs can form R-loops with the DNA that encodes them, thereby influencing local chromatin structure and gene expression by exposing single-stranded DNA regions or recruiting chromatin-modifying complexes [1]. The stability of triplexes is influenced by various factors, including pH, ionic strength, and the presence of divalent cations, such as magnesium. Recent studies have revealed that triplex formation can occur both in cis, where the lncRNA interacts with nearby genomic regions, and in trans, where the lncRNA targets distant genomic loci [19,20,21]. The biological significance of RNA–DNA triplexes is multifaceted. These structures can serve as molecular anchors, guiding lncRNAs to specific genomic locations where they can recruit protein complexes or modulate local chromatin structure [6]. For instance, the lncRNA MEG3 forms triplexes at the TGF-β pathway genes that include SMAD2, SMAD3, SMAD4, TGFB1, TGFB2, TGFB3, TGFBR1, and TGFBR2, recruiting chromatin remodeling complexes to regulate their expression [22] (Figure 1A). Triplex formation can also impact DNA replication and repair processes. The presence of R-loops can lead to replication fork stalling and genomic instability if not properly resolved [23]. Conversely, some lncRNAs may use triplex formation as a mechanism to protect certain genomic regions from DNA damage or to facilitate DNA repair processes [24]. The lncRNA TERRA, for example, forms triplexes at telomeric regions, potentially contributing to telomere maintenance and genome stability [25] (Figure 1B).

Recent technological advancements have greatly enhanced our ability to study RNA–DNA triplexes. Techniques such as Triplex-seq [26] and DNA–RNA immunoprecipitation sequencing (DRIP-seq) [27] allow for genome-wide identification of triplex-forming regions and R-loops, respectively. These approaches have revealed that triplex formation is more prevalent than previously thought and occurs at various genomic elements, including promoters, enhancers, and gene bodies. Enhancer-associated lncRNAs represent a subset of lncRNAs that are transcribed from enhancer regions and play critical roles in gene regulation. Enhancers are distal regulatory elements that can activate gene expression over long genomic distances [28]. The discovery that many enhancers are themselves transcribed to produce lncRNAs has added a new layer of complexity to our understanding of gene regulation [29]. Enhancer-associated lncRNAs can act as molecular scaffolds, recruiting transcription factors, coactivators, and chromatin modifiers to their sites of action. The lncRNA CCAT1-L, for instance, interacts with the CTCF protein to modulate chromatin looping at the MYC locus, influencing MYC expression in colorectal cancer cells [30] (Figure 1C). Similarly, the enhancer RNA (eRNA) NRIP1 facilitates the recruitment of cohesin and mediator complexes to estrogen-responsive enhancers, promoting estrogen-dependent gene activation [31,32].

The specificity and functionality of eRNAs are areas of ongoing research. While some eRNAs appear to have sequence-specific functions, others may act more generally through the process of transcription itself. The stability and processing of eRNAs also vary, with some being rapidly degraded while others are more stable and can even be spliced [33]. LncRNAs play a crucial role in shaping the three-dimensional organization of the genome, influencing everything from local chromatin accessibility to higher-order chromatin structures [16]. This process is fundamental to gene expression control and cellular identity. At the local level, lncRNAs can recruit chromatin-modifying complexes to specific genomic loci, leading to changes in histone modifications and DNA methylation status. The classic example of this is the Xist lncRNA, which coats the inactive X chromosome in female mammals, recruiting repressive complexes, like PRC1 and PRC2, that lead to chromosome-wide silencing [16,32]. Similarly, the HOTAIR lncRNA interacts with the PRC2 and LSD1 complexes to induce repressive chromatin states at the HOXD locus and other genomic regions [1,8].

LncRNAs also play crucial roles in the formation and maintenance of higher-order chromatin structures, such as topologically associating domains and chromatin loops. The FIRRE lncRNA, for instance, has been implicated in maintaining long-range chromosomal interactions between its site of transcription and other genomic loci, potentially contributing to the organization of nuclear architecture [6,34] (Figure 1D). The advent of high-resolution chromosome conformation capture techniques, such as Hi-C and ChIA-PET, combined with RNA-centric approaches like CHART and RAP, has provided unprecedented insights into the roles of lncRNAs in chromatin organization [32,35]. These studies have revealed that many lncRNAs are associated with specific chromatin interactions and can influence the formation of regulatory hubs within the nucleus. The interactions between lncRNAs and DNA represent a complex and dynamic aspect of gene regulation and genome organization. From the formation of triplex structures to the modulation of enhancer activity and the shaping of chromatin architecture, lncRNAs demonstrate remarkable versatility in their ability to influence DNA-based processes. As our understanding of these interactions continues to grow, so too does the potential for harnessing this knowledge for therapeutic interventions and biotechnological applications. Future research in this field promises to uncover even more intricate mechanisms by which lncRNAs contribute to the regulation of genomic function and cellular identity.

### 2.2. LncRNA–RNA Interactions

The intricate world of RNA–RNA interactions has emerged as a crucial aspect of gene regulation, with lncRNAs playing pivotal roles in modulating various RNA-mediated processes [36]. This section elucidates the multifaceted interactions between lncRNAs and other RNA species, focusing on three key areas: mRNA stability and translational control [37], miRNA sponge function [38], and splicing regulation [39]. These interactions highlight the remarkable versatility of lncRNAs in fine-tuning gene expression at the post-transcriptional level.

**Figure 2:**
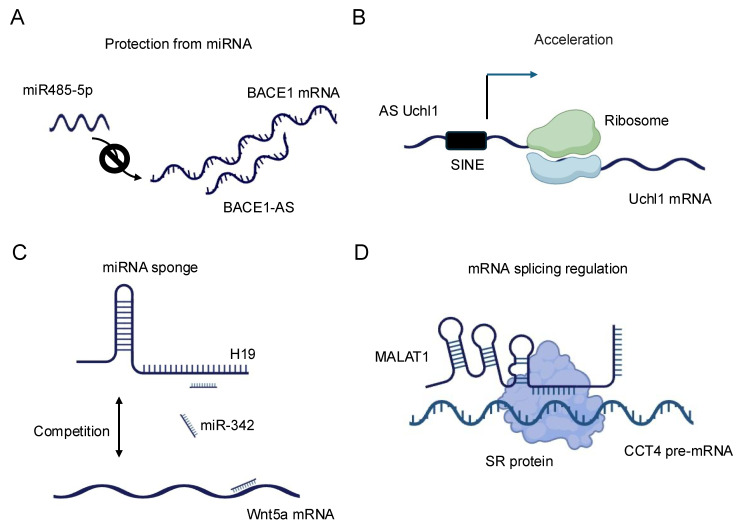
Schematic representation of the diverse regulatory functions of lncRNAs in gene expression. (**A**) BACE1-AS: BACE1 antisense RNA (BACE1-AS) protects BACE1 mRNA from microRNA (miRNA)-mediated degradation, thereby stabilizing BACE1 mRNA and enhancing its expression. (**B**) AS Uchl1: Antisense Uchl1 (AS Uchl1) accelerates the translation of Uchl1 mRNA, promoting increased protein synthesis. (**C**) H19: The lncRNA H19 modulates the expression of Wnt5a mRNA, illustrating its role in post-transcriptional gene regulation. (**D**) MALAT1: Metastasis-associated lung adenocarcinoma transcript 1 (MALAT1) regulates alternative splicing of pre-mRNAs, thereby influencing mRNA maturation and diversity.

The regulation of mRNA stability and translation by lncRNAs represents a sophisticated mechanism for controlling gene expression. LncRNAs can influence mRNA fate through direct base-pairing interactions or by modulating the activity of RNA-binding proteins that affect mRNA stability and translation efficiency [40]. A prominent example is the lncRNA BACE1-AS, which forms an RNA duplex with the BACE1 mRNA, increasing its stability and subsequently enhancing BACE1 protein levels [41] (Figure 2A). This interaction has implications in Alzheimer’s disease pathogenesis, underscoring the physiological relevance of such lncRNA–mRNA interactions. LncRNAs can also regulate mRNA stability by competing with or facilitating the binding of stabilizing or destabilizing factors to target mRNAs. For instance, the lncRNA NEAT1 has been shown to interact with and sequester the RNA-destabilizing protein KSRP, thereby indirectly stabilizing a subset of mRNAs involved in cancer progression [42]. Conversely, some lncRNAs can promote mRNA decay by recruiting deadenylation complexes or other decay-promoting factors to specific transcripts [36].

In terms of translational control, lncRNAs exhibit diverse mechanisms to modulate protein synthesis. Some lncRNAs can directly interact with ribosomes or translation initiation factors, influencing the efficiency of translation. The lncRNA AS Uchl1, for example, enhances the translation of the UCHL1 mRNA by facilitating its association with polysomes [43] (Figure 2B). Other lncRNAs may act as scaffolds, bringing together translational regulators and their target mRNAs to fine-tune protein synthesis in response to cellular cues [36]. Recent advances in high-throughput techniques, such as Cross-linking and Immunoprecipitation (CLIP)-seq [44] and ribosome profiling [45], have revealed the extensive involvement of lncRNAs in translational regulation. These studies have uncovered complex networks of lncRNA–mRNA interactions that respond to various cellular stresses and developmental signals, highlighting the dynamic nature of lncRNA-mediated translational control [37].

The miRNA sponge function of lncRNAs, also known as the competing endogenous RNA (ceRNA) mechanism, represents another fascinating aspect of lncRNA–RNA interactions. In this paradigm, lncRNAs can modulate the availability of miRNAs by acting as molecular decoys, thereby influencing the expression of miRNA target genes [46]. This mechanism adds an additional layer of complexity to the post-transcriptional regulatory network and has been implicated in various biological processes and disease states [38]. The concept of ceRNA activity was first prominently described with the discovery of the PTENP1 pseudogene, which acts as a decoy for miRNAs targeting the tumor suppressor PTEN [47]. Since then, numerous lncRNAs have been identified as miRNA sponges, with some capable of binding multiple miRNAs and thus potentially influencing entire gene expression programs. For example, the lncRNA H19 has been shown to sponge let-7 family miRNAs, affecting the expression of genes involved in muscle differentiation and cancer progression [48,49,50] (Figure 2C). The efficacy of lncRNAs as miRNA sponges depends on various factors, including the abundance of the lncRNA, the number and affinity of miRNA binding sites, and the cellular context. Recent studies have employed advanced bioinformatics approaches and experimental validations to identify bona fide ceRNA interactions. Techniques such as crosslinking, ligation, and sequencing of hybrids (CLASH) [51] and CLEAR–CLIP [52] have provided direct evidence for lncRNA–miRNA interactions in living cells. It is important to note that the physiological relevance of the ceRNA mechanism has been a subject of debate, with some studies suggesting that the abundance of most lncRNAs may be insufficient to significantly affect miRNA activity [50]. However, emerging evidence indicates that ceRNA effects may be particularly relevant in specific cellular compartments or under certain physiological conditions where the local concentrations of lncRNAs and miRNAs are sufficiently high [53].

The role of lncRNAs in splicing regulation represents yet another critical aspect of their RNA–RNA interactions. Alternative splicing is a key mechanism for generating proteome diversity and regulating gene expression, and lncRNAs have emerged as important modulators of this process [54]. LncRNAs can influence splicing decisions through various mechanisms, including direct interactions with pre-mRNAs, modulation of splicing factor activity, and alteration of chromatin structure at splice sites [55]. One well-characterized example of splicing regulation by lncRNAs is the MALAT1 lncRNA. MALAT1 localizes to nuclear speckles, where it interacts with and modulates the activity of several serine/arginine (SR) splicing factors (Figure 2D). By influencing the phosphorylation status and localization of these factors, MALAT1 can affect the splicing patterns of numerous pre-mRNAs, particularly those involved in cell cycle regulation and cancer progression [54]. Some lncRNAs can directly base pair with pre-mRNAs to mask or expose splice sites, thereby influencing splice site selection. The natural antisense transcript (NAT) Zeb2-AS, for instance, masks a 5′ splice site in the Zeb2 pre-mRNA, promoting intron retention and enhancing Zeb2 protein production [56]. This mechanism plays a crucial role in epithelial-mesenchymal transition, a process important in development and cancer metastasis. LncRNAs can also modulate splicing by altering the local chromatin environment around splice sites. The lncRNA NEAT1 plays a dual role in cellular processes [57]. It is critical for paraspeckle formation, where it acts as a scaffold by interacting with various proteins to assemble these nuclear bodies, which regulate gene expression under stress conditions. Additionally, NEAT1 influences alternative splicing by modulating the elongation kinetics of RNA polymerase II. This highlights how NEAT1 contributes to higher-order nuclear architecture and gene regulation. By promoting the formation of paraspeckles, NEAT1 can sequester certain splicing factors, indirectly affecting splicing decisions for a subset of genes.

The inclusion of circular RNA (circRNAs), intron-derived lncRNAs, and pseudogene-derived lncRNAs adds further complexity to the landscape of noncoding RNA biology. CircRNAs, due to their unique circular structure, are resistant to exonuclease-mediated degradation and often act as microRNA sponges or regulators of transcription. Intron-derived lncRNAs have been shown to modulate splicing events or interact with chromatin-modifying complexes. Transcribed pseudogenes, once considered genomic relics, are now recognized for their ability to act as ceRNAs or regulate parental gene expression through various mechanisms. The exploration of these subclasses provides new insights into the multifaceted roles of lncRNAs in gene regulation and disease.

Recent technological advancements, such as long-read sequencing [58] and single-cell RNA-seq [59], have provided unprecedented insights into the complexity of alternative splicing and the roles of lncRNAs in this process. These techniques have revealed extensive isoform diversity and cell-type-specific splicing patterns, many of which are influenced by lncRNAs. The diverse RNA–RNA interactions mediated by lncRNAs underscore their importance as key regulators of gene expression at the post-transcriptional level. From modulating mRNA stability and translation to acting as miRNA sponges and influencing splicing decisions, lncRNAs demonstrate remarkable versatility in fine-tuning RNA-based processes. As our understanding of these interactions continues to grow, so does the potential for developing RNA-based therapeutics and diagnostic tools. Future research in this field promises to uncover even more intricate mechanisms by which lncRNAs contribute to the complex tapestry of gene regulation and cellular function.

### 2.3. LncRNA–Protein Interactions

LncRNAs have emerged as crucial regulators of gene expression through their diverse interactions with proteins. These interactions are fundamental to understanding the molecular mechanisms by which lncRNAs exert their biological functions. This section explores three key aspects of lncRNA–protein interactions: their association with chromatin-modifying complexes, binding to transcription factors, and role in controlling the localization of macromolecules within the cell.

**Figure 3:**
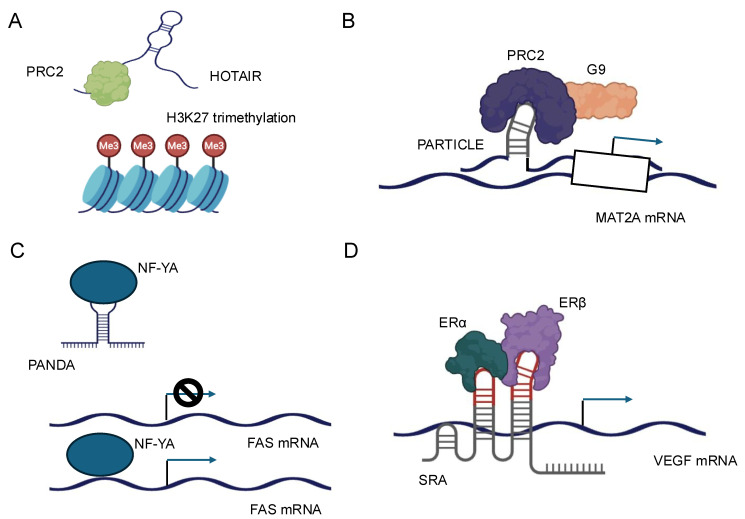
Schematic representation of the molecular mechanisms and cellular functions of four representative lncRNAs. (**A**) HOTAIR: HOTAIR acts as a scaffold for chromatin-modifying complexes, such as PRC2 and LSD1, leading to epigenetic gene silencing and regulation of gene expression. (**B**) PARTICLE: PARTICLE is involved in the regulation of gene expression in response to environmental stress, functioning through interactions with chromatin and modulation of transcriptional activity. (**C**) PANDA: PANDA interacts with transcription factors and regulatory proteins to inhibit apoptosis and promote cell survival, particularly in response to DNA damage. (**D**) SRA: SRA serves as a coactivator for nuclear receptors and other transcription factors, facilitating the regulation of gene transcription and influencing diverse cellular processes.

The interaction between lncRNAs and chromatin-modifying complexes represents a pivotal mechanism by which these noncoding transcripts influence gene expression at the epigenetic level [6,16]. LncRNAs can serve as molecular scaffolds, bringing together various components of chromatin-modifying complexes and guiding them to specific genomic loci. One of the most well-studied examples of this interaction is the association between lncRNAs and the Polycomb Repressive Complex 2 (PRC2) [16]. PRC2 is responsible for the trimethylation of histone H3 at lysine 27 (H3K27me3), a repressive epigenetic mark. Numerous lncRNAs, such as HOTAIR, have been shown to interact directly with PRC2 components, particularly EZH2, the catalytic subunit of the complex [16,60] (Figure 3A). These interactions facilitate the recruitment of PRC2 to specific genomic regions, leading to targeted gene silencing. The specificity of lncRNA-guided chromatin modification is often achieved through the formation of RNA–DNA triplex structures and through the recognition of specific DNA sequences by RNA-binding proteins associated with the lncRNA–chromatin modifier complex [26,32]. For instance, the lncRNA PARTICLE has been demonstrated to form triplexes at promoter regions of certain genes, influencing their expression in response to environmental stimuli, such as low-dose radiation [17] (Figure 3B).

Moreover, lncRNAs can interact with multiple chromatin-modifying complexes simultaneously, orchestrating complex patterns of histone modifications [32]. The lncRNA FENDRR, for example, has been reported to interact with both the MLL complex (associated with activating H3K4 methylation) and PRC2, allowing for fine-tuned regulation of target genes during embryonic development [61,62]. Recent technological advancements, such as Capture hybridization analysis of RNA targets (CHART) [63,64] and chromatin isolation by RNA purification (ChIRP) [65,66], have greatly enhanced our ability to map lncRNA–chromatin interactions genome-wide. These techniques have revealed that many lncRNAs associate with specific chromatin regions, often correlating with sites of active chromatin modification.

LncRNAs can modulate the activity of transcription factors through various mechanisms, including direct binding, sequestration, and alteration of post-translational modifications. These interactions can either enhance or inhibit the transcriptional activity of their protein partners, providing a nuanced control over gene expression. One mechanism by which lncRNAs regulate transcription factors is by acting as molecular decoys. In this scenario, lncRNAs can bind to transcription factors and sequester them away from their DNA targets, effectively inhibiting their transcriptional activity. For example, the lncRNA PANDA has been shown to interact with the transcription factor NF-YA, preventing it from activating pro-apoptotic genes following DNA damage [67] (Figure 3C).

Conversely, lncRNAs can also function as co-activators or co-repressors of transcription factors. By binding to transcription factors, lncRNAs can modulate their affinity for DNA, alter their interaction with other regulatory proteins, or influence their stability. The lncRNA SRA, for instance, acts as a co-activator for several nuclear receptors, including the estrogen receptor, enhancing their transcriptional activity [68] (Figure 3D). Furthermore, lncRNAs can influence the post-translational modifications of transcription factors, thereby affecting their activity or stability. Some lncRNAs have been found to interact with kinases or phosphatases that modify transcription factors, altering their phosphorylation status and, consequently, their transcriptional activity [69]. The subcellular localization of lncRNAs is a critical determinant of their function, and conversely, lncRNAs can influence the localization of their protein partners, affecting cellular processes and signaling pathways [70]. LncRNAs exhibit diverse subcellular localizations, including nuclear, cytoplasmic, and even organelle-specific distributions. This localization is often dynamic and can change in response to cellular stimuli or during different stages of development. The mechanisms governing lncRNA localization are complex and involve interactions with various RNA-binding proteins that recognize specific sequence or structural motifs within the lncRNA [64].

Nuclear retention of lncRNAs is often mediated by specific sequence elements or secondary structures that are recognized by nuclear retention factors. For example, the BORG lncRNA contains a SINE element that promotes its nuclear localization through interaction with the HNRNPK protein [71,72]. Conversely, lncRNAs can also influence the subcellular localization of proteins, particularly transcription factors and chromatin-modifying enzymes. By binding to these proteins, lncRNAs can either promote or inhibit their nuclear translocation, thereby regulating their access to genomic targets [57]. The lncRNA NRON, for instance, interacts with members of the importin-beta superfamily and the nuclear factor of activated T cells (NFAT), regulating the nuclear translocation of NFAT and subsequent transcriptional activity [73].

LncRNAs also play a role in the formation and maintenance of nuclear bodies, such as paraspeckles and nuclear speckles [74]. The interactions between lncRNAs and proteins, particularly in the context of chromatin modification, transcription factor regulation, and cellular localization control, represent a complex and dynamic aspect of gene regulation. As our understanding of these interactions continues to grow, so does the potential for developing novel therapeutic strategies targeting lncRNA–protein interactions in various diseases, including cancer and neurodegenerative disorders. Future research in this field promises to uncover even more intricate mechanisms by which lncRNAs contribute to the regulation of cellular function and organismal development.

Furthermore, HOTTIP exemplifies the integration of RNA–DNA, RNA–protein, and RNA–RNA interactions in gene regulation. It binds directly to chromatin at the HOXA gene cluster, recruiting the MLL1/WDR5 complex to activate HOXA gene transcription through histone modifications. HOTTIP also interacts with epigenetic regulators, like MLL1 and DOT1L, modifying chromatin structure and regulating gene expression, as seen in rheumatoid arthritis, where it recruits MLL1 to the TLR4 promoter. Additionally, HOTTIP functions as a competing endogenous RNA, sponging microRNAs, such as miR-19a-3p and miR-19b-3p, to regulate cancer progression pathways, and interacting with miRNAs, like miR-216a, in small cell lung cancer.

## 3. LncRNA in Biomolecular Condensates

LncRNAs play key roles in the formation and regulation of biomolecular condensates, which are dynamic assemblies of proteins and nucleic acids involved in various cellular processes. They mediate phase separation, nuclear body formation, and the assembly of transcriptional control hubs.

The capacity of lncRNAs to promote phase separation is rooted in their distinctive molecular characteristics. In contrast to their protein-coding counterparts, lncRNAs exhibit intrinsic structural flexibility and the ability to form complex secondary and tertiary structures [75]. These attributes enable lncRNAs to interact simultaneously with multiple protein partners, often through low-complexity domains or intrinsically disordered regions. Such multivalent interactions are fundamental to the process of liquid–liquid phase separation (LLPS), which underlies the formation of biomolecular condensates [76,77,78,79].

LncRNAs function as scaffolds or nucleators for phase separation, providing a platform for the assembly of proteins and other nucleic acids. Their length and ability to form specific structural motifs enable them to create a network of interactions that can lower the critical concentration required for phase separation [80,81,82]. The formation of nuclear bodies represents another critical aspect of lncRNA function in biomolecular condensates. Nuclear bodies are membrane-less structures within the nucleus that concentrate specific sets of proteins and RNAs to perform specialized functions. LncRNAs play pivotal roles in nucleating and maintaining these structures [83,84,85]. In addition to paraspeckles, lncRNAs are involved in the formation of other nuclear bodies, such as nuclear stress bodies and histone locus bodies [84]. Nuclear stress bodies, for example, are formed in response to various cellular stresses and are nucleated by the lncRNA HSATIII [85]. This lncRNA is rapidly transcribed upon heat shock and serves as a platform for the recruitment of specific splicing factors and other RNA-binding proteins. The formation of these bodies is hypothesized to regulate gene expression and RNA processing during stress conditions [86,87]. A recent groundbreaking discovery in this field is the identification of a novel nuclear structure called the Heat-inducible Noncoding RNA-containing nuclear body (HiNoCo body) [88]. This structure forms in response to heat shock and contains the lncRNA MALAT1, which relocates from nuclear speckles. HiNoCo bodies are distinct from other known nuclear bodies, form independently of HSF1, and show reversible dynamics. Cells lacking MALAT1 exhibited reduced proliferation after heat shock, suggesting a crucial role for HiNoCo bodies in stress response.

The construction of transcriptional control hubs represents another crucial function of lncRNAs within biomolecular condensates. These hubs are specialized condensates that bring together various regulatory elements, transcription factors, and co-factors to control gene expression. LncRNAs can serve as organizational centers for these hubs, facilitating the assembly of complex regulatory networks [26]. One mechanism by which lncRNAs contribute to these hubs is through their ability to interact with both DNA and proteins. Some lncRNAs can form RNA–DNA triplexes, allowing them to target specific genomic loci while simultaneously recruiting regulatory proteins [17]. This bridging function enables lncRNAs to create three-dimensional regulatory landscapes within the nucleus. By sequestering these factors, lncRNAs can fine-tune gene expression patterns. The lncRNA NORAD, for instance, sequesters PUMILIO proteins in the cytoplasm, preventing them from repressing their target mRNAs and thereby maintaining genomic stability [89,90]. The involvement of lncRNAs in biomolecular condensates represents a paradigm shift in our understanding of gene regulation and cellular organization. Through their roles in promoting phase separation, forming nuclear bodies, and constructing transcriptional control hubs, lncRNAs emerge as master regulators of cellular processes. As research in this field progresses, it is becoming increasingly evident that the unique properties of lncRNAs make them ideally suited to orchestrate the complex, dynamic assemblies that underlie many aspects of cellular function. Future investigations will likely uncover even more intricate mechanisms by which lncRNAs shape the physical and functional landscape of the cell through their interactions within biomolecular condensates.

## 4. Structural Basis of LncRNA Interactions

The structural basis of lncRNA interactions is a critical aspect in elucidating their diverse functions in cellular processes. LncRNAs exhibit unique structural features that facilitate interactions with various biomolecules, including DNA, RNA, and proteins, thereby influencing gene regulation, chromatin organization, and cellular signaling pathways.

LncRNAs form complex secondary and tertiary structures, which are essential for their biological functions. Compared to mRNAs, lncRNAs are more structured but less complex than ribosomal RNAs [91,92,93]. This intermediate organization provides a balance between flexibility and stability, supporting their diverse roles. LncRNAs frequently possess distinct structural motifs that contribute to their overall architecture. These motifs encompass stem-loops, hairpins, bulges, internal loops, and pseudoknots [94] The combination and arrangement of these structural elements create a unique three-dimensional landscape that facilitates specific interactions with other molecules. For instance, the lncRNA HOTAIR contains multiple stem-loop structures that are essential for its interaction with the PRC2 complex, a key player in epigenetic regulation [95] (Figure 3A). Chemical probing experiments, such as Selective 2′-Hydroxyl Acylation analyzed by Primer Extension (SHAPE), have provided valuable insights into the secondary structure of lncRNAs [96]. These studies have demonstrated that many lncRNAs form hierarchical structures with distinct subdomains, each containing multiple secondary structure motifs. This hierarchical organization allows lncRNAs to interact with multiple partners simultaneously and perform complex regulatory functions [97,98].

The modular structure of lncRNAs is a fundamental aspect of their functionality. Many lncRNAs are composed of discrete functional domains that can operate independently or in concert to achieve specific biological outcomes [99]. These modules often correspond to distinct secondary structure elements or clusters of motifs that serve as interaction platforms for proteins or other nucleic acids [95,100]. The modular nature of lncRNAs confers functional versatility and evolutionary plasticity. Different modules can be acquired, lost, or rearranged over evolutionary time, leading to the diversification of lncRNA functions across species [7]. This modular architecture also enables lncRNAs to act as molecular scaffolds, bringing together multiple protein complexes to form functional ribonucleoprotein assemblies [90,101]. Functional domains in lncRNAs can range from a few nucleotides to hundreds of bases in length. These domains can mediate specific interactions with proteins, such as chromatin modifiers or transcription factors, or facilitate base-pairing with other nucleic acids [33]. For example, the lncRNA NEAT1 contains distinct domains that are responsible for its role in paraspeckle formation and its interaction with specific RNA-binding proteins [57].

The significance of repetitive sequences in lncRNA structure and function cannot be overstated. Repetitive elements, once considered “junk DNA”, are now recognized as critical components of many lncRNAs. These repeats can be derived from transposable elements, simple sequence repeats, or tandem duplications of functional motifs [102]. Repetitive sequences in lncRNAs serve multiple purposes. They can act as structural building blocks, creating regular patterns that contribute to the overall architecture of the RNA. For instance, the MALAT1 lncRNA contains a triple helix structure at its 3′ end, formed by repetitive sequences, which protects it from degradation and influences its cellular localization [103]. Moreover, repeats can function as protein-binding platforms, allowing for the recruitment of multiple copies of the same protein or different proteins to a single lncRNA molecule. This property is particularly important for lncRNAs that act as molecular scaffolds or participate in the formation of membrane-less organelles through phase separation [104,105]. The evolutionary conservation of repetitive elements in lncRNAs across species suggests their functional importance. While the primary sequence of lncRNAs often evolves rapidly, the presence and arrangement of certain repetitive motifs can be conserved, indicating selective pressure to maintain these structural features [102].

The structural complexity imparted by repetitive sequences allows lncRNAs to fine-tune their interactions with various cellular components [106]. Some repeats can serve as spacers between functional domains, providing the necessary flexibility for lncRNAs to adopt different conformations in response to cellular conditions or binding partners [106,107]. The structural basis of lncRNA interactions is a multifaceted aspect of their biology, encompassing unique structural features, modular organization, and the critical role of repetitive sequences. The complex secondary and tertiary structures formed by lncRNAs provide a diverse landscape for molecular interactions, enabling these versatile molecules to perform a wide array of cellular functions. As our understanding of lncRNA structure continues to advance, so does our appreciation for the intricate relationship between their architecture and their biological roles. Future research in this field promises to elucidate even more sophisticated mechanisms by which lncRNAs leverage their structural properties to regulate gene expression, modulate chromatin states, and orchestrate cellular processes.

My analysis of existing literature reveals a compelling need to explore the functional redundancy among lncRNAs and their compensatory mechanisms in gene regulation. I propose that the evolutionary conservation of lncRNA secondary structures, rather than primary sequences, may hold the key to understanding their specificity in biomolecular interactions. This perspective aligns with our ongoing research into RNA structure–function relationships, which suggests that structural motifs could serve as universal platforms for protein recruitment across diverse biological contexts.

## 5. Methods to Study LncRNA Interactions

The investigation of lncRNA interactions has become increasingly crucial in elucidating their diverse roles in cellular processes. This section explores the various methodologies employed to analyze RNA structure, identify interaction partners, and elucidate the functions of lncRNAs. Despite the fact that lncRNAs are typically expressed at very low abundance in cells, these experimental approaches have been optimized to successfully investigate these molecules, making all these sophisticated analyses feasible even with limited starting material.

RNA structure analysis techniques have advanced significantly, providing researchers with powerful tools to investigate the complex architectures of lncRNAs. Chemical and enzymatic probing approaches, such as SHAPE and Dimethyl Sulfate sequencing (DMS-seq), utilize chemical modifications of RNA nucleotides to reveal structural information [93,108,109]. These techniques have been adapted for high-throughput analysis, enabling transcriptome-wide structural studies. A particularly innovative method in RNA structure analysis is in vivo click SHAPE with mutational profiling (icSHAPE–MaP) [110]. This technique combines the advantages of icSHAPE reagents with mutational profiling in reverse transcription, allowing for the probing of intact RNA structures in living cells. icSHAPE–MaP has been successfully applied to determine the complete structural information for cellular small RNAs and can be combined with RNA immunoprecipitation to study the structural landscape of specific RNA–protein complexes [111].

Another cutting-edge approach is fragmentation sequencing (Frag-Seq), which employs nuclease P1 to specifically degrade single-stranded nucleic acids. This method, followed by high-throughput sequencing and bioinformatic analysis, provides valuable insights into RNA secondary structures on a genome-wide scale [112]. The development of long-read sequencing technologies has further enhanced the ability to capture complex RNA structures and isoforms. Computational methods play a crucial role in complementing experimental approaches for RNA structure analysis. Advanced algorithms and machine learning techniques are being developed to predict RNA secondary and tertiary structures based on sequence information and experimental data [113]. These computational tools are particularly valuable for studying large lncRNAs, where experimental methods may have limitations. Identifying interaction partners of lncRNAs is essential for understanding their molecular functions. A variety of techniques have been developed to study lncRNA–protein, lncRNA–DNA, and lncRNA–RNA interactions. For lncRNA–Protein interactions, methods such as RNA immunoprecipitation (RIP) followed by sequencing (RIP-seq) have been widely used [114,115]. These techniques allow for the identification of proteins associated with specific lncRNAs in vivo.

Cross-linking and immunoprecipitation (CLIP) and its variants such as High-Throughput Sequencing of RNA isolated by Crosslinking Immunoprecipitation (HITS–CLIP) [44] and Photoactivatable-Ribonucleoside-Enhanced Crosslinking and Immunoprecipitation (PAR–CLIP) [116] provide higher resolution by identifying the precise binding sites of proteins on lncRNAs. These techniques involve UV crosslinking to stabilize RNA–protein interactions, followed by immunoprecipitation and high-throughput sequencing. Moreover, RNA And DNA interacting complexes ligated, and sequence (RAD–ICL-seq) involves cross-linking RNA, proteins, and DNA in nuclei using formaldehyde, then linking RNA and DNA via adapters. After removing proteins, complexes are sequenced to map interactions genome-wide, revealing both known and novel RNA-chromatin structures [117]. RADIP extends RADICL-seq by incorporating immunoprecipitation to specifically enrich RNA–DNA complexes associated with target proteins. This involves cross-linking, adapter-mediated ligation, and antibody-based purification, followed by sequencing to comprehensively map protein-mediated DNA–RNA interactions genome-wide [118].

For studying lncRNA–DNA interactions, techniques such as Capture Hybridization Analysis of RNA Targets (CHART) and Chromatin Isolation by RNA Purification (ChIRP) have been developed [65,119] (Table 1). These methods use biotinylated oligonucleotides complementary to the lncRNA of interest to pull down associated chromatin, allowing for the identification of genomic binding sites. To investigate lncRNA–RNA interactions, methods like CLASH and psoralen analysis of RNA interactions and structures (PARIS) have been employed [51,120] (Table 2). These techniques can reveal direct base-pairing interactions between lncRNAs and other RNA molecules, providing insights into potential regulatory mechanisms. Recent advancements in proximity ligation-based methods, such as mapping RNA interactome in vivo (MARIO), have enabled the study of RNA–RNA interactions on a genome-wide scale [120]. These techniques can capture both direct and indirect interactions, offering a comprehensive view of the RNA interactome.

Functional analysis approaches for lncRNAs have become increasingly sophisticated, allowing researchers to elucidate their roles in various biological processes. The CRISPR-Cas9 system and its derivatives, such as CRISPR interference (CRISPRi) and CRISPR activation (CRISPRa), have been adapted for lncRNA studies, enabling targeted repression or activation of lncRNA expression from their endogenous loci [121,122]. High-throughput CRISPR screens have been developed specifically for lncRNAs, allowing for the simultaneous interrogation of hundreds or thousands of lncRNAs [123,124]. These screens can be designed to identify lncRNAs involved in specific cellular processes, such as proliferation, drug resistance, or differentiation [122]. Antisense oligonucleotides (ASOs) and small interfering RNAs (siRNAs) continue to be valuable tools for lncRNA knockdown studies, especially for lncRNAs that are difficult to target with CRISPR-based approaches [125]. Locked nucleic acid GapmeR (LNA GapmeRs), a type of ASO, have shown promise in targeting nuclear lncRNAs, which are often resistant to siRNA-mediated knockdown. For gain-of-function studies, overexpression of lncRNAs using traditional plasmid-based systems or more advanced methods like CRISPRa can reveal phenotypes associated with increased lncRNA levels [126]. The choice between plasmid-based overexpression and CRISPRa depends on the specific research question, as each method has its advantages and limitations. Emerging technologies, like RNA-guided RNA-targeting CRISPR effectors (e.g., Cas13), are opening new avenues for lncRNA functional studies. These systems offer the potential for highly specific RNA targeting in both the nucleus and cytoplasm, although their utility is still being fully explored.

The field of lncRNA research is rapidly evolving, with new methods continually being developed to study their structure, interactions, and functions. The integration of multiple approaches, including high-throughput sequencing, advanced imaging techniques, and computational analysis, is crucial for gaining a comprehensive understanding of lncRNA biology. As these methods continue to improve and new technologies emerge, our knowledge of lncRNA functions and their roles in health and disease will undoubtedly expand, potentially leading to new therapeutic strategies and diagnostic tools.

## 6. Biological Significance and Disease Relevance

In the development and differentiation, lncRNAs exhibit intricate and stage-specific expression patterns that are essential for proper organismal development. During embryogenesis, numerous lncRNAs are expressed in a highly regulated manner, contributing to the complex gene regulatory networks that guide cell fate decisions and tissue patterning. For instance, the lncRNA Braveheart has been demonstrated to play a critical role in cardiovascular lineage commitment [127]. This lncRNA interacts with the PRC2 complex to epigenetically regulate key cardiac genes, illustrating how lncRNAs can influence cell fate through chromatin modification. In the nervous system, lncRNAs, such as Evf2 and Pnky, are involved in neuronal differentiation and the maintenance of neural stem cell populations [128,129]. Evf2 functions as a co-activator for the Dlx transcription factors, which are crucial for GABAergic interneuron development, while Pnky regulates neural stem cell differentiation through modulation of alternative splicing. These examples highlight the diverse mechanisms by which lncRNAs can influence developmental processes.

LncRNAs also play significant roles in maintaining stem cell pluripotency and directing lineage-specific differentiation. The lncRNA RMST, for example, is essential for neuronal differentiation of human embryonic stem cells [130]. It interacts with SOX2, a key pluripotency factor, to co-regulate neurogenic genes [131]. In the hematopoietic system, lncRNAs such as HOTAIRM1 are involved in myeloid differentiation, demonstrating the importance of lncRNAs in blood cell development [132]. LncRNAs are deeply involved in disease processes, especially cancer. They contribute to tumor initiation, progression, metastasis, and drug resistance. For example, MALAT1 is overexpressed in several cancers and promotes tumor growth and metastasis by regulating gene expression and alternative splicing [133]. Another well-studied oncogenic lncRNA, HOTAIR, is associated with increased metastasis and poor prognosis in several cancers [134]. Hypoxia-induced HIF-1α also upregulates lncRNA STEAP3-AS1, which competitively interacts with YTHDF2 to enhance STEAP3 mRNA stability and protein expression. This STEAP3-AS1/STEAP3/Wnt/β-catenin axis promotes colorectal cancer (CRC) progression, offering potential diagnostic biomarkers and therapeutic targets for CRC treatment [135]. Conversely, some lncRNAs act as tumor suppressors. The lncRNA MEG3, for example, inhibits tumor growth by activating p53 and repressing the MDM2 oncogene [136]. The loss of MEG3 expression has been observed in various cancer types, underscoring its importance in maintaining cellular homeostasis. These examples illustrate how lncRNAs can function as both oncogenes and tumor suppressors, depending on their specific molecular interactions and cellular context.

Beyond cancer, lncRNAs have been implicated in a wide range of other diseases, including neurodegenerative diseases. In neurodegenerative disorders, such as Alzheimer’s disease, the lncRNA BACE1-AS regulate the expression of genes involved in amyloid-β production, potentially contributing to disease pathogenesis [41]. The involvement of lncRNAs in various diseases has naturally led to interest in their potential as therapeutic targets. The unique properties of lncRNAs, including their tissue-specific expression and diverse regulatory mechanisms, make them attractive candidates for targeted therapies. Several approaches are being explored to modulate lncRNA function for therapeutic purposes [137]. One promising strategy is the use of ASOs to target specific lncRNAs. ASOs can be designed to bind to lncRNAs and induce their degradation or alter their secondary structure, thereby modulating their function. This approach has shown promise in preclinical studies for targeting oncogenic lncRNAs like MALAT1 [138]. Another method involves the use of siRNAs to knockdown lncRNA expression. While this approach has been successful in vitro, delivery challenges remain for in vivo applications. CRISPR-Cas9 technology is also being explored for lncRNA-based therapies. This approach allows for precise genomic editing to modulate lncRNA expression or function. Small molecule inhibitors that target specific lncRNA–Protein interactions are another avenue being explored for therapeutic intervention. These molecules can disrupt the binding of lncRNAs to their protein partners, potentially altering disease-associated pathways [139]. However, identifying small molecules that specifically target RNA–Protein interactions remains challenging and is an active area of research (Table 3).

LncRNAs also hold promise as diagnostic and prognostic biomarkers [140,141]. Their tissue-specific expression patterns and presence in bodily fluids make them attractive candidates for non-invasive diagnostics. For instance, the lncRNA PCA3 is already used clinically as a biomarker for prostate cancer detection [142]. Similarly, circulating lncRNAs in blood or other biofluids are being investigated as potential biomarkers for various cancers and other diseases [143]. The biological significance of lncRNAs in development, differentiation, and disease processes is becoming increasingly evident. Their diverse roles in regulating gene expression, chromatin structure, and cellular signaling pathways position them as important players in both normal physiology and pathological conditions. As our understanding of lncRNA biology continues to expand, so does the potential for developing lncRNA-based therapies and diagnostic tools [144]. However, significant challenges remain, including improving our understanding of lncRNA structure–function relationships, developing effective delivery methods for RNA-based therapeutics, and elucidating the complex regulatory networks in which lncRNAs participate. Overcoming these challenges will be crucial for translating our knowledge of lncRNA biology into effective clinical applications.

## 7. Advances in Technologies for Exploring lncRNA Functions

For single-cell and spatial transcriptomics mapping of lncRNA activity, recent work has demonstrated how these advanced technologies are revealing cell-type specific and spatially distinct expression patterns of lncRNAs in various tissues, particularly in the context of cancer and neurodegenerative diseases [145,146,147]. For example, studies have analyzed single-cell and spatial transcriptomics data to uncover novel lncRNAs across multiple cancer types and map lncRNA spatial expression in colorectal cancer [145]. These approaches are providing unprecedented insights into the complex regulatory roles of lncRNAs within heterogeneous tissue environments. Regarding advances in cryo-EM revealing 3D structural insights into lncRNA complexes, recent protocols and methodological improvements have optimized RNA sample preparation for cryo-EM analysis [148,149,150]. These developments have enhanced the ability to resolve high-resolution structures of lncRNAs and their interaction partners. Additionally, cryo-electron tomography is now being applied to study RNA structures in their native cellular contexts, offering a powerful approach to understand lncRNA function within the complex intracellular milieu [148]. Emerging RNA-centric CRISPR tools, particularly Cas13 systems, have shown great promise for RNA knockdown and editing, as well as therapeutic applications [151]. Recent studies have elucidated the characteristics of various Cas13 effectors (Cas13a-i), highlighting their unique properties and potential uses. For instance, RfxCas13d has demonstrated the ability to target RNA without requiring a protospacer flanking sequence, expanding the range of potential RNA targets [151]. These advances in RNA-targeting CRISPR technologies are opening new avenues for studying lncRNA function and developing RNA-based therapeutics.

## 8. Conclusions and Future Perspectives

Recent years have seen remarkable advancements in the field of lncRNA research, revolutionizing our understanding of gene regulation and cellular function. This comprehensive review has explored the multifaceted nature of lncRNAs, from their structural characteristics to their diverse roles in biological processes and disease pathogenesis. As I conclude, it is essential to synthesize key findings, address unresolved questions, and outline future research directions in this rapidly evolving field. One of the most significant insights from lncRNA studies is their versatility in regulating gene expression. LncRNAs function at multiple levels, including transcriptional regulation, post-transcriptional processing, and epigenetic modification. Their ability to interact with DNA, RNA, and proteins enables them to serve as molecular scaffolds, guides, and decoys, orchestrating complex regulatory networks within cells. The discovery of lncRNAs involved in phase separation and biomolecular condensate formation has expanded our understanding of their role in cellular organization and function [106]. Recent research has focused on the structural basis of lncRNA interactions. Advanced RNA structure analysis techniques have revealed that lncRNAs possess complex secondary and tertiary structures crucial for their function. The modular nature of many lncRNAs, with distinct functional domains and repetitive elements, provides a flexible platform for diverse molecular interactions [91]. Their structural complexity enables precise interactions with biomolecules and dynamic responses to cellular conditions. In development and differentiation, lncRNAs are critical regulators of cell fate decisions and tissue-specific gene expression programs. From embryonic stem cell maintenance to lineage-specific differentiation, lncRNAs play essential roles in fine-tuning gene expression networks. Their involvement in processes such as X chromosome inactivation and genomic imprinting highlights their importance in fundamental biological phenomena [61]. The implication of lncRNAs in various diseases, particularly cancer, has driven the field forward. Numerous lncRNAs have been identified as oncogenes or tumor suppressors, influencing key hallmarks of cancer, such as proliferation, metastasis, and drug resistance. Beyond cancer, lncRNAs have been implicated in cardiovascular diseases, neurodegenerative disorders, and autoimmune conditions, underscoring their broad relevance to human health [133]. Despite these advances, several challenges remain. One major challenge is the functional characterization of the vast number of lncRNAs identified through high-throughput sequencing. Developing efficient strategies to systematically investigate their functions is a pressing need. Another challenge lies in understanding how lncRNAs achieve specificity in their interactions with other biomolecules. Elucidating the structural and sequence determinants governing these interactions will be crucial for predicting lncRNA functions and designing targeted therapies.

The evolutionary conservation of lncRNAs remains a topic of debate. While some lncRNAs show high sequence conservation across species, many others exhibit poor sequence conservation but maintain structural or functional conservation. Developing better computational and experimental approaches to assess lncRNA conservation will be important for understanding their biological roles and translating findings from model organisms to humans. In therapeutics, significant hurdles remain in translating our knowledge of lncRNAs into effective clinical interventions. Challenges include developing efficient delivery methods for RNA-based therapeutics, ensuring specificity of action, and minimizing off-target effects [137]. A deeper understanding of their tissue-specific roles is necessary before they can be safely targeted in a therapeutic context. Looking to the future, several promising research directions are emerging. One area of interest is the further exploration of lncRNA roles in phase separation and biomolecular condensate formation [42]. Understanding how lncRNAs contribute to the organization of these membrane-less organelles could provide new insights into both normal physiology and disease processes. The integration of lncRNA research with emerging fields like single-cell genomics and spatial transcriptomics holds great promise [145]. These technologies will allow for a nuanced understanding of lncRNA expression and function at the individual cell level and within complex tissues. Such approaches could reveal cell type-specific functions of lncRNAs and their roles in maintaining cellular heterogeneity within tissues. Advances in structural biology techniques, including cryo-electron microscopy and nuclear magnetic resonance spectroscopy, will provide unprecedented insights into lncRNA structure and interactions [147]. Combining these structural data with functional studies will be crucial for understanding the molecular mechanisms underlying lncRNA function and designing targeted therapeutic interventions. The development of sophisticated CRISPR-based tools for lncRNA manipulation will enable more precise functional studies [121]. Techniques allowing for the modulation of lncRNA expression or structure without altering the underlying DNA sequence will be particularly valuable for dissecting lncRNA functions in their native genomic context.

Finally, translating lncRNA research into clinical applications remains a key goal. This includes not only developing lncRNA-based therapeutics but also utilizing lncRNAs as diagnostic and prognostic biomarkers. The tissue-specific expression patterns of many lncRNAs make them attractive candidates for non-invasive diagnostics, and further research could lead to novel clinical tools [142]. The study of lncRNAs has dramatically expanded our understanding of gene regulation and cellular function. As we continue to unravel their complex roles in development, disease, and cellular organization, new opportunities for therapeutic intervention and diagnostic applications are likely to emerge. The future of lncRNA research promises to be both challenging and exciting, with the potential to fundamentally reshape our understanding of biology and medicine.

Building upon the current understanding of lncRNA biology, several bold, testable hypotheses emerge that warrant future exploration. I propose that lncRNAs function as master structural organizers of nuclear architecture, with specific structural motifs determining their capacity to scaffold nuclear bodies and regulate chromatin territories in a cell type-specific manner [16]. This hypothesis could be tested through systematic structure-function analyses of lncRNAs involved in nuclear organization, coupled with high-resolution imaging of chromatin dynamics. Furthermore, I anticipate that the dynamic interplay between lncRNA structure and phase separation properties directly modulates gene expression programs during cellular differentiation and stress response [42], which could be investigated using in vitro reconstitution systems combined with single-cell transcriptomics. Looking forward, I envision significant paradigm shifts through the integration of lncRNA studies with emerging fields. The convergence of lncRNA biology with epi-transcriptomics presents an exciting frontier, as RNA modifications likely influence lncRNA structure, stability, and protein-binding capabilities, thereby expanding their regulatory repertoire [87]. Similarly, integrating lncRNA research with 3D genomics will provide unprecedented insights into how these molecules orchestrate genome folding and nuclear compartmentalization. These interdisciplinary approaches may reveal that lncRNAs serve as critical nodes in cellular information processing networks, coordinating responses to environmental stimuli through their ability to simultaneously interact with chromatin, proteins, and other RNA species. Such paradigm shifts will not only deepen our understanding of fundamental biological processes but also open new avenues for therapeutic interventions targeting lncRNA-mediated nuclear organization in disease states.

In conclusion, lncRNAs represent a fascinating frontier in molecular biology, with implications spanning development, disease, and therapeutic innovation. Expanding upon the postulation that chromatin restructuring facilitated by lncRNAs plays a fundamental role in cellular adaptive responses, subsequent research ought to focus on structural investigations with enhanced resolution and comprehensive functional evaluations to illuminate these regulatory pathways [34,35]. By integrating these insights, we aim to contribute to a deeper understanding of lncRNA biology and its translational potential.

## Figures and Tables

**Table 1:** Methods for Analyzing RNA–DNA Interactions.

Method	Mechanism	Key Applications
CHART	Uses hybridization probes to capture specific RNA and identify associated DNA regions and protein complexes.	Identification of DNA regions and protein complexes associated with specific RNAs.
CHIRP	Isolates chromatin regions associated with specific RNAs using biotin-labeled probes. Potential to treat a wide range of genetic conditions	High-resolution mapping of chromatin regions bound by specific RNAs.
RADICL-seq	Detects RNA–DNA proximal interactions and ligates interacting complexes for sequencing.	Genome-wide analysis of RNA-chromatin interactions.
Triplex-seq	Analyzes RNA functioning as triplex structures with DNA on chromatin.	Investigation of RNA–DNA triplex structures and their roles in chromatin regulation.

**Table 2:** Methods for Analyzing RNA–Protein Interactions.

Method	Mechanism	Key Applications
CLASH	Combines crosslinking, ligation, and sequencing to identify both RNA–Protein and RNA–RNA interactions.	Simultaneous mapping of RNA–Protein and RNA–RNA interaction networks.
HITS-CLIP	Employs UV crosslinking to map direct RNA–Protein interaction sites at high resolution.	High-resolution mapping of RNA–Protein binding sites.
MARIO	Maps RNA interactome networks in vivo, focusing on RNA–RNA interactions.	Comprehensive analysis of RNA–RNA interaction networks in living cells.
PAR-CLIP	Utilizes photoactivatable ribonucleosides for efficient crosslinking and mapping of RNA-binding proteins (RBPs) binding sites.	Enhanced identification of RNA–Protein interaction sites compared to HITS-CLIP.
RIP-seq	Identification of RNAs bound to specific RNA-binding proteins (RBPs).	Identification of RNAs bound to specific RBPs.

**Table 3:** LncRNAs and Their Involvement in Diseases.

lncRNA Name	Involvedment in Diseases	Key Funstion
MEG3	Malignant tumors, cardiovascular diseases, metabolic diseases, immune system diseases	Acts as a tumor suppressor; regulates p53 signaling; influences apoptosis, autophagy, and inflammation.
TERRA	Aging, telomeropathies, immunodeficiency syndromes	Regulates telomere homeostasis; interacts with telomeric proteins; implicated in aging and immune responses.
CCAT1-L	Colorectal cancer, gastric adenocarcinoma	Promotes MYC expression via chromatin looping; facilitates tumor proliferation, invasion, and metastasis.
FIRRE	Colorectal cancer, diffuse large B-cell lymphoma, gallbladder cancer	Enhances proliferation and migration; regulates inflammatory genes and autophagy through RNA–Protein interactions.
BACE1-AS	Alzheimer’s disease, heart failure	Regulates BACE1 expression; promotes β-amyloid production; potential biomarker for AD progression.
AS Uchl1	Parkinson’s disease, Alzheimer’s disease	Enhances UCHL1 translation via SINEUP mechanism; neuroprotective role in neurodegenerative diseases.
MALAT1	Lung cancer, asthma, pulmonary fibrosis, rheumatoid arthritis	Regulates cell proliferation and migration; acts as a miRNA sponge; potential therapeutic target in lung diseases.
H19	Cancer, cardiovascular diseases	Functions as a miRNA sponge; regulates gene expression in tumor progression and cardiac remodeling.
PARTICLE	Cardiovascular diseases	Interacts with PRC2 to regulate chromatin structure and gene expression.
FENDRR	Cardiovascular diseases, pulmonary fibrosis	Modulates FOXF1 expression via PRC2 interactions; involved in embryonic development.
PANDA	Cancer	Protects against DNA damage by interacting with NF-YA transcription factor.
SRA	Breast cancer, cardiovascular diseases	Regulates steroid receptor activity; influences apoptosis and angiogenesis.

## Data Availability

No new data were created.
